# An electronic portfolio for quantitative assessment of surgical skills in undergraduate medical education

**DOI:** 10.1186/1472-6920-13-65

**Published:** 2013-05-06

**Authors:** Serafín Sánchez Gómez, Elisa María Cabot Ostos, Juan Manuel Maza Solano, Tomás Francisco Herrero Salado

**Affiliations:** 1 Department of Otorhinolaryngology, Virgen Macarena University Hospital, C/Fernández de Rivera 16, B, 9° D, Seville 41005, Spain; 2 University of Seville, Av. Dr. Fedriani 3, Seville 41071, Spain; 3 Department of Surgery, University of Seville, Av. Dr. Fedriani 3, Seville 41071, Spain

**Keywords:** Electronic portfolio, Surgical subjects, Self-guided learning, Self-assessment, Evaluative portfolio

## Abstract

**Background:**

We evaluated a newly designed electronic portfolio (e-Portfolio) that provided quantitative evaluation of surgical skills. Medical students at the University of Seville used the e-Portfolio on a voluntary basis for evaluation of their performance in undergraduate surgical subjects.

**Methods:**

Our new web-based e-Portfolio was designed to evaluate surgical practical knowledge and skills targets. Students recorded each activity on a form, attached evidence, and added their reflections. Students self-assessed their practical knowledge using qualitative criteria (yes/no), and graded their skills according to complexity (basic/advanced) and participation (observer/assistant/independent). A numerical value was assigned to each activity, and the values of all activities were summated to obtain the total score. The application automatically displayed quantitative feedback. We performed qualitative evaluation of the perceived usefulness of the e-Portfolio and quantitative evaluation of the targets achieved.

**Results:**

Thirty-seven of 112 students (33%) used the e-Portfolio, of which 87% reported that they understood the methodology of the portfolio. All students reported an improved understanding of their learning objectives resulting from the numerical visualization of progress, all students reported that the quantitative feedback encouraged their learning, and 79% of students felt that their teachers were more available because they were using the e-Portfolio. Only 51.3% of students reported that the reflective aspects of learning were useful. Individual students achieved a maximum of 65% of the total targets and 87% of the skills targets. The mean total score was 345 ± 38 points. For basic skills, 92% of students achieved the maximum score for participation as an independent operator, and all achieved the maximum scores for participation as an observer and assistant. For complex skills, 62% of students achieved the maximum score for participation as an independent operator, and 98% achieved the maximum scores for participation as an observer or assistant.

**Conclusions:**

Medical students reported that use of an electronic portfolio that provided quantitative feedback on their progress was useful when the number and complexity of targets were appropriate, but not when the portfolio offered only formative evaluations based on reflection. Students felt that use of the e-Portfolio guided their learning process by indicating knowledge gaps to themselves and teachers.

## Background

Portfolio-based assessment tool is at the apex of Miller’s pyramid, because it provides performance-based assessment in real context by analysis of actions [1,2]. Portfolios are widely replacing logbooks in medical education. Logbooks are simple collections of tasks performed but do not include critical reflections, and recording of activities in a logbook may be viewed as a chore rather than a way to stimulate learning. Portfolios include critical reflection, and therefore encourage performance and learning as a challenge in a way that logbooks do not [3]. Undergraduate students usually complete logbooks that describe the diseases they should observe [4], but these do not usually include tasks to develop practical skills. Reflection is a metacognitive process that creates a greater understanding of both the self and the situation, so that future actions can be influenced by this understanding [5]. Although there is little research evidence to suggest that reflection improves quality of care, it may enhance the care process [6].

Buckley et al. [7] reported that the strength and extent of evidence supporting the educational effectiveness of using portfolios in the undergraduate setting are limited. However, their selected “higher quality” papers tended to show that portfolio use was associated with improved knowledge and understanding, increased self-awareness, engagement in reflection, and improved student-teacher relationships. They also indicated that although portfolios encourage students to engage in reflection, the quality of this cannot be assumed, and the time commitment required to complete portfolios may detract from other learning activities or deter students from engaging in the process unless required to do so for assessment. These authors, and other authors, consider that further evaluation of the usefulness of portfolios is needed, particularly comparative studies that directly observe changes in student knowledge and abilities, rather than simply reporting on student perceptions after completion of a portfolio [8].

There is ongoing debate regarding the relative learning impacts of electronic portfolios versus paper-based portfolios [9]. Electronic portfolios can provide immediate feedback [10], and can use hyperlinks to organize material and link to relevant content and objectives. They can enhance learning by providing organizational flexibility, flexibility in the presentation of content and ideas, and links to other sources and other forms of representation [11]. Electronic portfolios can also collect and display evidence of learning from many sources such as texts, presentations, images, photographs, and videos, that students can upload and access using devices such as smartphones, tablets, and laptops.

Most of the currently used portfolios are designed primarily for formative assessment and focused on the learning process. They present students with information about their strengths and weaknesses, and help to formulate plans for improvement [12]. These portfolios promote assessment of generic skills, but offer limited quantitative feedback on the acquisition of specific skills [13]. A few portfolios rate progress using summative assessments [14]. Properly designed portfolios should allow students to assess their own learning, and should provide a record of training outcomes. The aim of the present study was to evaluate the usefulness to students of our newly designed electronic portfolio (e-Portfolio) in terms of self-guidance and self-evaluation for the learning of specific skills. The e-Portfolio was used as a replacement for the current system of evaluation which is based simply on the presence of students in different clinical settings for a specified number of hours [15], without quantification of their performance [16]. In addition to providing formative assessments, the portfolio provided automatic quantitative assessments of skill acquisition or improvement by displaying numerical indications of progress [17].

## Methods

The new e-Portfolio was used to evaluate students taking undergraduate surgical courses at the University of Seville. The e-Portfolio was web-based and was available online via all standard web browsers [18]. The database was designed to be accessible via the web environment at the University of Seville (http://www.portfolioelectronico.com/cirugiayorlsevilla). The application was simple and intuitive, and was designed for users with a low to medium level of experience with information and communication technologies. Access to the application was by individual username and password. Pilot studies of the e-Portfolio exceeded the requirements for robustness proposed by Barrett [19], and did not detect problems with the interface, missing links, blank pages, missing pages, incorrect algorithms, or incorrect feedback.

Practical knowledge and skills targets were selected from the University of Seville Undergraduate Otorhinolaryngology Programme, and were adapted to meet the requirements of the European Space for Higher Education [20]. This program specifies the learning objectives that students should achieve during the mandatory hours of practice. Achievement of the objectives was planned in terms of “deliberate practice” [21]. The objectives were grouped into knowledge targets to achieve knowledge regarding patient management, and skills targets to achieve specific abilities (Table 1).

**Table 1:** Knowledge targets

	**Outpatients**	**Self-evaluation**	**Subtotal**
**1**	Otorhinolaryngology outpatient office	0/1	0/9
**2**	Apparatus, instruments and pharmacology. Hand washing	0/1	
**3**	Otorhinolaryngology medical record	0/1	
**4**	Complementary studies	0/1	
**5**	Preoperative evaluation	0/1	
**6**	Operated patients evaluation	0/1	
**7**	Post-surgery protocols: pharmacology, cures, laboratory studies	0/1	
**8**	Patient information and Informed Consent	0/1	
**9**	Otorhinolaryngology documents	0/1	
	**Hospitalization**		
**1**	Otorhinolaryngology hospitalization ward	0/1	0/13
**2**	Otorhinolaryngology medical record	0/1	
**3**	Complementary studies	0/1	
**4**	Preoperative evaluation	0/1	
**5**	Patient preparation for surgery. Antibiotic prophylaxis. Hematologic prophylaxis	0/1	
**6**	Apparatus, instruments	0/1	
**7**	Emergencies	0/1	
**8**	Surgical patients management	0/1	
**9**	Wound care	0/1	
**10**	Administration of drugs	0/1	
**11**	Drains and drain management	0/1	
**12**	Surgery ward security protocols	0/1	
**13**	Diets, fluid therapy	0/1	
	**Operating room**		
**1**	Otorhinolaryngology operating room	0/1	0/10
**2**	Professional behavior in an otorhinolaryngology operating room	0/1	
**3**	Sterility, surgical hand washing, gowns/cap/mask/gloves placement	0/1	
**4**	Patient preparation for surgery: position, personnel, and apparatus	0/1	
**5**	Apparatus, instruments and pharmaceuticals	0/1	
**6**	Surgical site preparation: sterile drapes, antiseptic techniques	0/1	
**7**	Sutures: materials, types, mechanisms, and procedures	0/1	
**8**	Operating room security protocols	0/1	
**9**	Operating room documents	0/1	
**10**	Surgical techniques: fundamentals, indications, procedures	0/1	

Qualitative self-evaluations (0/1), and the subtotals of each section.

The e-Portfolio included 32 knowledge targets, including knowledge about outpatient units (9 targets), knowledge about hospitalized patients (13 targets), and knowledge about operating rooms (10 targets). Prior to software conversion, these targets were displayed in the first column of a table. The second column showed whether each objective had been achieved (yes/no, translated to 0/1 on a numerical scale). A cumulative score of at least 26 points (>80%) was required before students were considered to have sufficient knowledge. The Department of Surgery set this cutoff figure to ensure that future physicians would be competent in most of the targeted objectives.

The e-Portfolio also included 20 skills targets, which were divided into basic abilities (8 targets) and advanced abilities (12 targets). Each skills target could be achieved by different degrees of participation as an observer, assistant, or independent operator. The skills were taught in clinical settings, and performance was directly observed by the teachers (Table 2). The e-Portfolio displayed these targets in the first column of a table. This table also included columns indicating the number of times that the student participated in an activity as an observer, assistant, or independent operator, and columns that weighted each type of participation. The sum of the weighted activity scores was recorded for each target. Minimum and maximum scores were set for each target, to enable competition among students and record progress as a percentage of required learning.

**Table 2:** Skills targets

		**Number of activities**			**Weight**			**Progress**	**Minimum score**	**Maximum score**
		**A**_**(no)**_	**B**_**(na)**_	**C**_**(ni)**_	**W**_**o**_	**W**_**a**_	**W**_**i**_			
**1**	Non-Surgical hand washing				Ax1	Bx2	Cx3	∑A:B:C	5	15
**2**	Otorhinolaryngology patient assessment				Ax1	Bx2	Cx3	∑A:B:C	5	15
**3**	Otorhinolaryngology medical record				Ax1	Bx2	Cx3	∑A:B:C	5	15
**4**	Application forms for additional evidence				Ax1	Bx2	Cx3	∑A:B:C	5	15
**5**	Patient information. Informed consent				Ax1	Bx2	Cx3	∑A:B:C	5	15
**6**	Surgical hand washing				Ax1	Bx2	Cx3	∑A:B:C	5	15
**7**	Sterility: gown/cap/mask/gloves placement				Ax1	Bx2	Cx3	∑A:B:C	5	15
**8**	Sterility: placement of surgical drapes, antisepsis				Ax1	Bx2	Cx3	∑A:B:C	5	15
**9**	Nasogastric tubes				Ax2	Bx3	Cx4	∑A:B:C	15	25
**10**	Cervical drains				Ax2	Bx3	Cx4	∑A:B:C	15	25
**11**	Wound care				Ax2	Bx3	Cx4	∑A:B:C	15	25
**12**	Otoscopy				Ax2	Bx3	Cx4	∑A:B:C	15	25
**13**	Rhinoscopy				Ax2	Bx3	Cx4	∑A:B:C	15	25
**14**	Oropharyngoscopy				Ax2	Bx3	Cx4	∑A:B:C	15	25
**15**	Balance and equilibrium				Ax2	Bx3	Cx4	∑A:B:C	15	25
**16**	Management of epistaxis				Ax2	Bx3	Cx4	∑A:B:C	15	25
**17**	Ear wash				Ax2	Bx3	Cx4	∑A:B:C	15	25
**18**	Acumetry				Ax2	Bx3	Cx4	∑A:B:C	15	25
**19**	Audiometry				Ax2	Bx3	Cx4	∑A:B:C	15	25
**20**	Removal of nasal foreign body				Ax2	Bx3	Cx4	∑A:B:C	15	25
	**Score range**								**220**	**420**

Participation in activities as an observer [n_o_], assistant [n_a_], or independent operator [n_i_], weighting of activities, and minimum and maximum scores.

**Figure 1:**
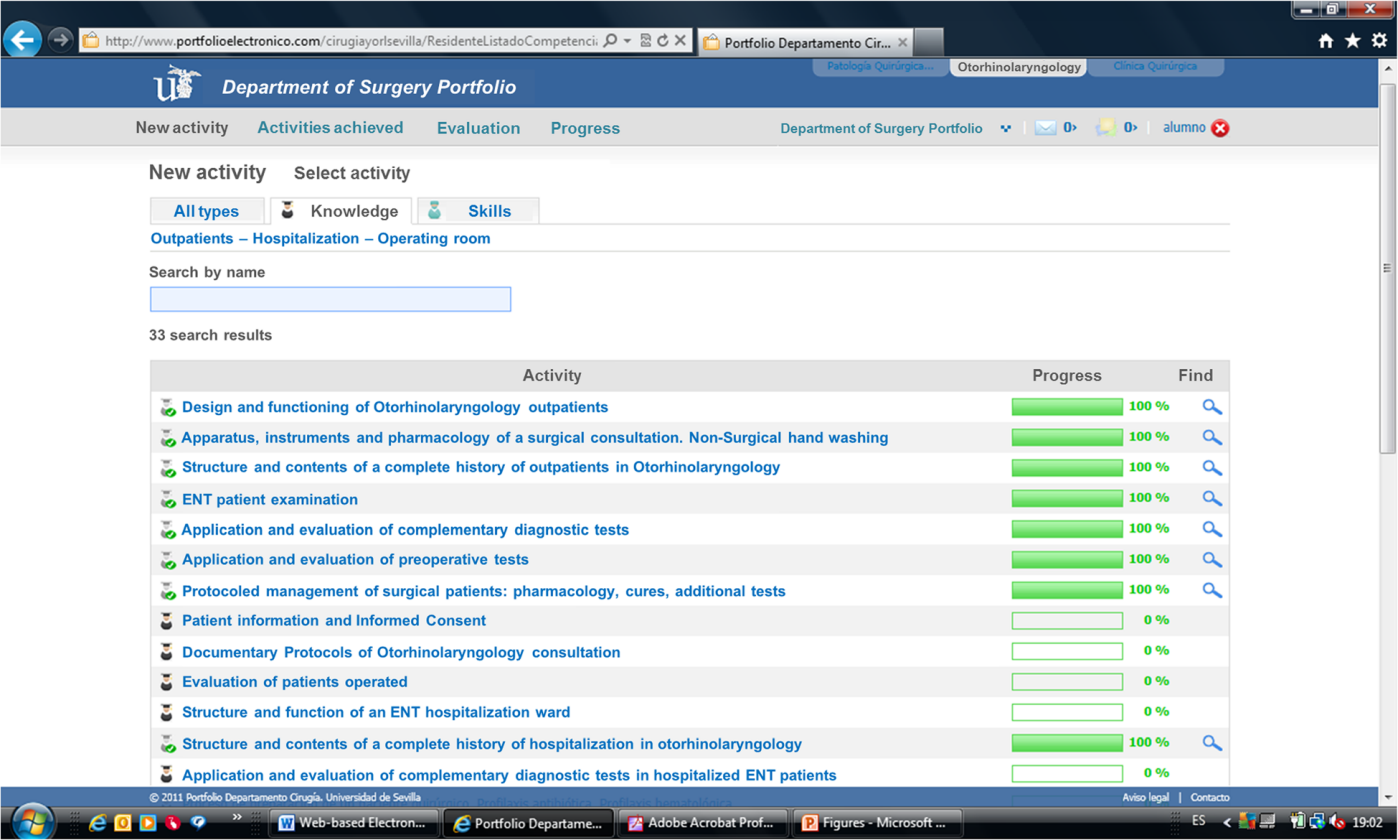
Screenshot of KNOWLEDGE NEW ACTIVITY page, showing the quantitative evaluation of progress.

**Figure 2:**
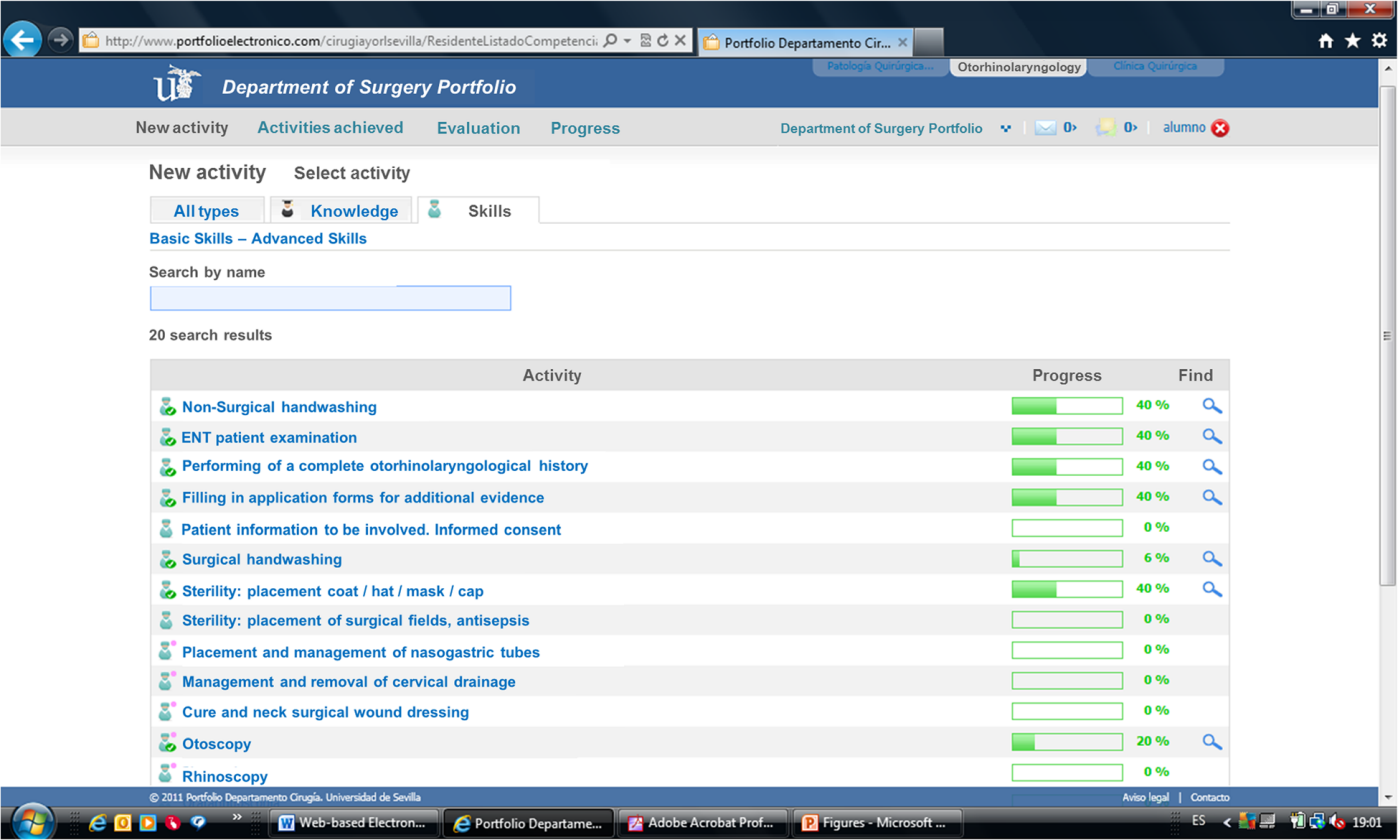
**Screenshot of NEW ACTIVITY form.** Students clicked the SAVE button to increase their scores and get automatic feedback.

**Figure 3:**
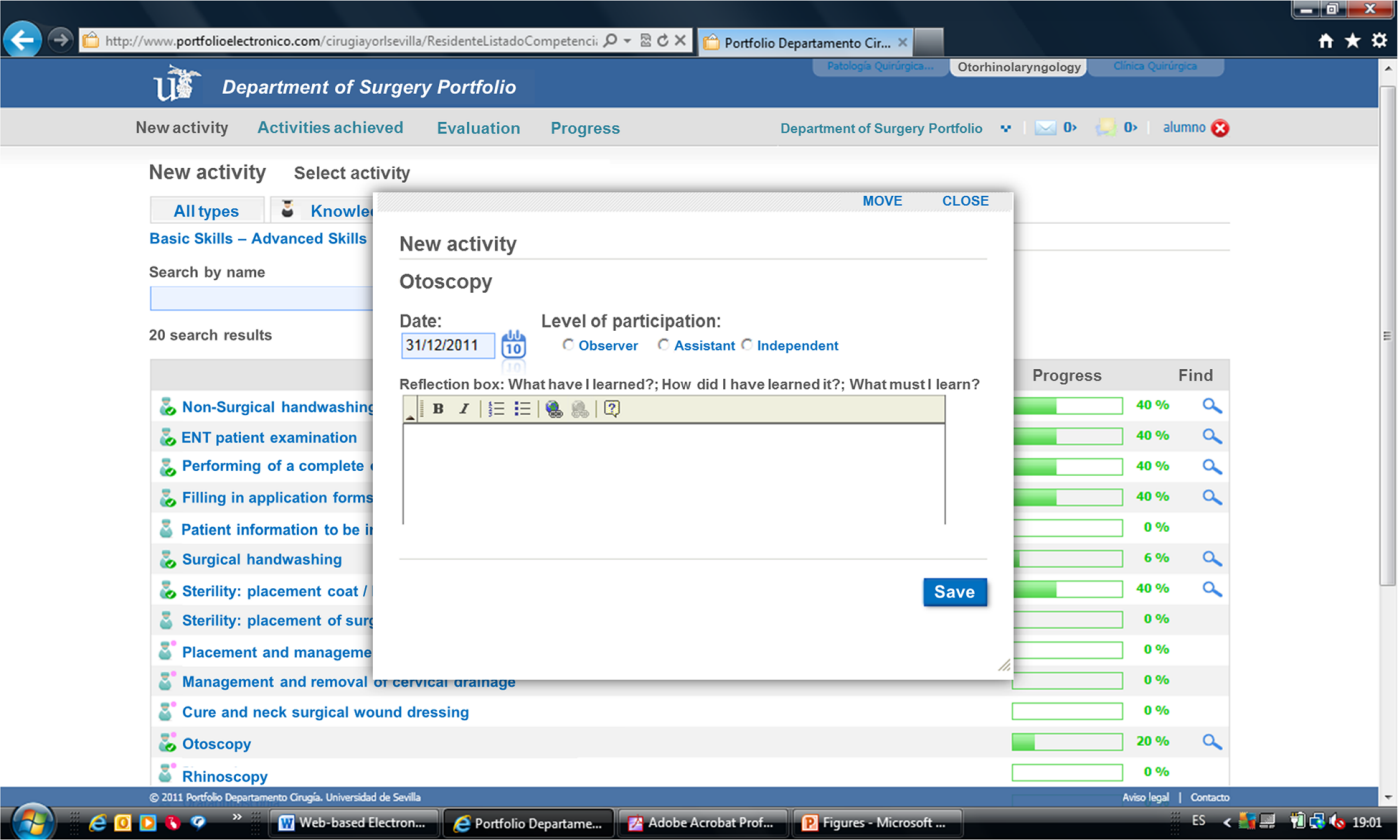
Screenshot of ABILITY NEW ACTIVITY page.

The e-Portfolio provided visual indications of progress towards achieving the knowledge and skills targets (Figures 1 and 2). The program also indicated the objectives that would be achieved with the completion of each target. Students registered completion of knowledge activities using the form that appeared when clicking on the corresponding target. The form included an area for free text input to record observations regarding competence or personal experience, including reflections on “what did I learn?”, “how did I learn it?”, and “what else should I learn?”. Students registered completion of skills activities using a different form (Figure 3) that recorded the type of participation for each activity: observer, assistant, or independent operator.

As the e-Portfolio allowed user data files to be imported and exported, self-assessments could be completed at various times and using various computers. Evidence of activities was attached to the forms and sent to the teachers for validation, including photographs, presentations, videos, or text files [22]. The e-Portfolio provided automatic numerical feedback regarding the progress of each student, and informed students of their outstanding targets. Students could use this feedback to achieve new activities ant help motivate further progress. Minimum and maximum scores were set for each activity to encourage students to complete a number of activities for each target. For each skills target, students were required to register participation as an observer at least five times, and could register participation as an independent operator a maximum of five times. Students were considered to have achieved the objectives when they had a minimum score of 220, provided they had also achieved the minimum score for each skill, and could score a maximum of 420 points if they participated in each activity five times as an independent operator. The e-Portfolio automatically assigned 1 point for each activity for which students had written more than 10 words in the free text field, reserving assessment by the teacher for the final evaluation. Activities were recorded in the database when the student clicked the “save” button. The teachers validated the evidence and provided feedback on their formative evaluations, regardless of the automatic numerical feedback provided by the e-Portfolio.

The e-Portfolio updated students on their activities and training progress, including both overall progress and specific progress in each learning area, using filters that each user was able to set individually. The e-Portfolio automatically sent students feedback when evidence was submitted and validated, presented as a percentage of the required achievements in both text and image (icon) formats. The e-Portfolio also provided a summary of each student’s training and learning progress on a page that allowed students to upload a personal photograph. The visibility of the photograph increased as the student progressed through the learning targets. Students were also able to print their portfolio in Excel® file format.

The e-Portfolio was described to the 112 medical students who were trained in the Otorhinolaryngology Department at Virgen Macarena University Hospital, Seville, Spain during 2010–2011. Use of the e-Portfolio was voluntary. We evaluated the usefulness of the portfolio to students at the end of the academic year using a structured questionnaire, and by randomly selecting 16 students for semi-structured interviews that followed the same structure as the questionnaire. We evaluated students’ understanding of the portfolio methodology, understanding of the learning targets, ease of use of the e-Portfolio, interest in learning surgical subjects, and perceived usefulness of the e-Portfolio in terms of learning outcomes. The roles of clinical teachers were reduced to teaching skills in clinical settings, to allow students to guide their own learning and evaluations. Data were analyzed using the triangulation technique. The usefulness of the e-Portfolio was evaluated by measuring the proportions of learning targets achieved.

## Results

Of the 112 students, 37 (33%) agreed to use the e-Portfolio to register their learning activities, using a variety of electronic devices. When asked about their understanding of the methodology of the portfolio, 87% of students said they understood the methodology and 12% were able to explain the methodology precisely when questioned. Many students (79%) reported that the teachers were more willing to help them achieve the learning targets after they were made aware of the learning model of the e-Portfolio. All students who used the e-Portfolio reported that they gained a better understanding of the objectives of the course by focusing on the target activities. They appreciated the reduction of the vast field of possible diagnoses and operations to a small number of targets that seemed achievable. The focus on a manageable number of targets allowed them to determine what they needed to learn during the course.

Nineteen students (51.3%) reported that reflective writing in the free text fields was useful, but that they had not written much because they did not know what to write. Another 13 students (35.1%) reported that they had written something because they expected that the teacher would take this into account, but they thought it was a waste of time. Five students (13.5%) reported that they considered written observations to be useless. Seventeen students (46%) asked their teachers to focus on the targets listed in the portfolio, thereby reducing more general teaching and teaching of material that was not included as a target.

Seventeen students (46%) reported that they had initially worked eagerly on the e-Portfolio because they had found it an interesting initiative, but eventually felt disenchanted because other students and some teachers did not show much interest. These students also felt that because the e-Portfolio was a temporary and voluntary activity, it would not be useful for longer term evaluations.

All students agreed that the quantification of their performance using numerical feedback was the most valuable aspect of the e-Portfolio. They felt that the e-Portfolio model was more useful for learning practical skills than the previous model that simply required students to be present in clinical settings and then tested them later.

Individual students achieved a maximum of 65% of the overall targets and 87% of the skills targets. The mean total score was 345 ± 38 points. For basic skills, 92% of students achieved the maximum score for participation as an independent operator, and all achieved the maximum scores for participation as an observer and assistant. For complex skills, 62% of students achieved the maximum score for participation as an independent operator, and 98% achieved the maximum scores for participation as an observer or assistant.

The evidence provided by students consisted mainly of photographs and videos recorded during activities by their classmates using their smartphones, and the files were uploaded to the e-Portfolio using their laptops later.

## Discussion

The portfolio model remains relatively unknown among clinical teachers and students. Disappointing experiences reported by students (time consuming, low expectations in terms of learning, dependence on the interest of mentors, poor willingness to participate) and teachers (time consuming, poor knowledge of methodology) have hindered the implementation of this model [23]. Awareness regarding the use of electronic portfolios is particularly low. Since the launching of the Urban Universities Portfolio Project (UUPP) [24], institutional electronic portfolios have started to gain popularity. However, successful experiences with electronic portfolios in Spanish medical settings, such as the electronic portfolio of the Spanish Society of Otorhinolaryngology for training graduate otorhinolaryngology residents [25], have not been transferred to the university setting.

It is likely that so few students in this study chose to use the e-Portfolio because of a lack of knowledge and experience regarding this learning model. Although the methodology was well understood when explained, few students (12%) were able to describe it precisely when questioned. The voluntary nature of inclusion in the study also reduced participation, as some students perform only mandatory activities, or activities that will be reviewed and evaluated. The current educational culture discourages written self-assessment of learning, resulting in a widespread resistance and lack of interest in producing written observations. The electronic portfolio model that was developed by Virgen Macarena University Hospital was probably particularly useful by combining into a single portfolio information that is usually found in separate portfolios [26]. Our model combined registration of activities with updates on progress regarding achievement of the learning targets [27]. The e-Portfolio model is more likely to be useful if it is a mandatory learning activity.

Considering current technology, access to an electronic portfolio via the internet should be considered standard [28]. Many devices now provide internet access, including smartphones, tablets, and laptops. Our students usually recorded photographs and videos using their smartphones, and then uploaded them using their laptops. Future versions of the e-Portfolio will allow direct uploading of such files via smartphones.

The most important feature of the e-Portfolio was the requirement for students to complete a predetermined number of target activities. Students were able to improve their skills by repeating activities with their clinical teachers in ways that paid attention to specific aspects of the tasks. All activities were assigned a numerical value, and the values of all activities were summated to obtain the total score. Observation of the progress of the total score increased interest in completing complex activities. Progress was recorded without testing, so that formative assessment was integrated with the numerical evaluation, without being punitive.

The e-Portfolio allowed students to understand their learning objectives using specific targets, rather than using the previous generic objectives [29]. Students were therefore able to direct their own learning. This was demonstrated by 17% of students requesting their teachers to focus on the learning targets presented in the e-Portfolio.

Reflective portfolios do not usually provide quantitative evaluations of activities and are teacher-dependent tools that require teacher assessments as well as student observations [30]. The e-Portfolio is mainly student-dependent, and is scored according to participation in activities and automatic numerical teacher-independent feedback. The e-Portfolio differs from the logbooks widely used in postgraduate training [31] as it provides both a record of activities and an indication of progress towards completion of the learning targets as a percentage. Students and teachers are therefore kept updated on progress and on outstanding requirements [32], which provides more encouragement than use of a logbook.

Numerical scores may give some students a false sense of security that they will pass the course, without encouraging identification of their shortcomings [33]. However, we found that visualization of the increasing scores as new targets were achieved encouraged students to continue learning. Use of the e-Portfolio resulted in some students trying to achieve higher scores by performing more activities. The e-Portfolio also provided a text field for reflective input that was assessed by the teachers at the end of the course. We were therefore able to include both numerical and formative types of assessment in our unique learning tool.

Students generally preferred to complete the skills targets than the knowledge targets. This may be because they were interested in the new teaching and learning experiences and the feedback provided by the e-Portfolio. Both students and teachers were encouraged to increase the roles of students in achieving their learning targets. This was a remarkable difference compared with the rejection of purely reflective portfolios.

The limitations of this first version of the e-Portfolio include its primary focus on achieving skills targets, rather than on a more comprehensive assessment of competence including communication skills, knowledge, technical skills, clinical reasoning skills, emotional skills, values, and observations that would benefit patients as well as society [34,35]. However, our experience shows that students can be more dynamic, active, demanding, flexible, autonomous, critical, and responsible when they are supported by an appropriate learning tool. It is expected that further development of the e-Portfolio will improve the achievement of competence by use of this unique combination of quantitative and formative assessments [36,37].

## Conclusions

Medical students found that use of an electronic portfolio that provided both quantitative feedback on their progress and formative evaluations stimulated their learning. The students felt that use of the e-Portfolio provided valuable guidance to their learning process by indicating their progress towards achievement of the skills targets, and that this motivated both them and their teachers to focus more clearly on the learning objectives. Identification of an appropriate number and complexity of learning targets helped the students and their teachers to evaluate progress and outstanding objectives.

## Competing interests

The Authors declare that they have no competing interests. The Electronic Portfolio of the Faculty of Medicine was supported by a financial fund from the 2009 Innovation and Teaching Enhancing Programme, University of Seville, Spain. The manuscript processing charges were financed by the Andalusian Association for Medical Education in Otorhinolaryngology.

## Authors’ contributions

SSG conceived of the study, participated in study design and coordination, drafted the manuscript, and performed the statistical analyses. JMOB and EMCO participated in study design, helped to analyze the results, and helped to draft the manuscript. All authors read and approved the final manuscript.

## Authors’ information

SSG participated in conceiving, coordinating, and implementing the Electronic Portfolio of the Spanish Society of Otorhinolaryngology and Cervical-Facial Pathology (named FORMIR^©^). This is a nationwide electronic portfolio for formative and summative assessment of all Spanish otorhinolaryngology residents. The portfolio was presented at An International Association for Medical Education (AMEE) 2010 in Glasgow, Scotland, and at the First Congress of the Iberoamerican Academy of Otorhinolaryngology 2011 in Cancun, Mexico. SSG is a member of the Spanish National Committee of Otorhinolaryngology, which is responsible for the National Programme of Otorhinolaryngology. SSG is also President of the Andalusian Foundation for Research and Knowledge Management in Otorhinolaryngology, President of the Andalusian Association for Medical Education in Otorhinolaryngology, and Secretary of the Andalusian Association for Health Sciences Education.

SSG, JMOB, and EMCO received grants from the University of Seville through several Learning Innovation projects including Electronic Portfolios, Problem Based Learning, and Learning Research.

## Ethical approval

No ethical approval was required for this study according to Spanish laws. However, the Faculty of Medicine at the University of Seville reviewed and approved the study protocol and carefully considered the related ethical issues. Anonymity of participants was guaranteed, and no potential harm to participants was identified.

## Pre-publication history

The pre-publication history for this paper can be accessed here:

http://www.biomedcentral.com/1472-6920/13/65/prepub
